# Antibacterial and cytotoxic cytochalasins from the endophytic fungus *Phomopsis* sp. harbored in *Garcinia kola* (Heckel) nut

**DOI:** 10.1186/s12906-016-1454-9

**Published:** 2016-11-14

**Authors:** Jean-Bosco Jouda, Jean-de-Dieu Tamokou, Céline Djama Mbazoa, Clovis Douala-Meli, Prodipta Sarkar, Prasanta Kumar Bag, Jean Wandji

**Affiliations:** 1 Department of Organic Chemistry, Faculty of Science, University of Yaoundé 1, P.O. Box 812, Yaoundé, Cameroon; 2 Department of Biochemistry, Faculty of Science, Laboratory of Microbiology and Antimicrobial Substances, University of Dschang, P.O. Box 67, Dschang, Cameroon; 3 Department of Biochemistry, University of Calcutta, 35 Ballygunge Circular Road, Kolkata, 700 019 India; 4 Julius Kühn Institut, Federal Research Centre for Cultivated Plants, Institute for National and International Plant Health, Messeweg 11-12, D-38104 Braunschweig, Germany

**Keywords:** *Garcinia kola*, Endophytic fungi, *Phomopsis* sp, Metabolites, Cytochalasins, Antibacterial, Cytotoxic

## Abstract

**Background:**

The continuous emergence of multidrug-resistant (MDR) bacteria drastically reduced the efficacy of our antibiotic armory and consequently, increased the frequency of therapeutic failure. The search for bioactive constituents from endophytic fungi against MDR bacteria became a necessity for alternative and promising strategies, and for the development of novel therapeutic solutions. We report here the isolation and structure elucidation of antibacterial and cytotoxic compounds from *Phomopsis* sp*.*, an endophytic fungus associated with *Garcinia kola* nuts.

**Methods:**

The fungus *Phomopsis* sp*.* was isolated from the nut of *Garcinia kola.* The crude extract was prepared from mycelium of *Phomopsis* sp*.* by maceration in ethyl acetate and sequentially fractionated by column chromatography. The structures of isolated compounds were elucidated on the basis of spectral studies and comparison with published data. The isolated compounds were evaluated for their antibacterial and anticancer properties by broth microdilution and 3-(4,5-dimethylthiazol-2-yl)-2,5-diphenyltetrazolium bromide methods respectively. The samples were also tested spectrophotometrically for their hemolytic properties against human red blood cells.

**Results:**

The fractionation of the crude extract afforded three known cytochalasins including 18-metoxycytochalasin J (1), cytochalasins H (2) and J (3) together with alternariol (4). The cytochalasin compounds showed different degrees of antibacterial activities against the tested bacterial pathogens. *Shigella flexneri* was the most sensitive microorganism while *Vibrio cholerae* SG24 and *Vibrio cholerae* PC2 were the most resistant. Ampicillin did not show any antibacterial activity against *Vibrio cholerae* NB2, *Vibrio cholerae* PC2 and *Shigella flexneri* at concentrations up to 512 μg/mL, but interestingly, these multi-drug resistant bacterial strains were sensitive to the cytochalasin metabolites. These compounds also showed significant cytotoxic properties against human cancer cells (LC_50_ = 3.66–35.69 μg/mL) with low toxicity to normal non-cancer cells.

**Conclusion:**

The three cytochalasin compounds isolated from the *Phomopsis* sp*.* crude extract could be a clinically useful alternative for the treatment of cervical cancer and severe infections caused by MDR *Shigella* and *Vibrio cholerae.*

## Background

Endophytic fungi are organisms that live inside the plant tissues and behave as plant hosts [1]. They have proven to be a rich source of novel organic compounds with interesting biological activities and a high level of biodiversity [2, 3]. Natural products from endophytic fungi have been observed to inhibit or kill a wide variety of harmful microorganisms including phytopathogens, as well as bacteria, fungi, viruses, and protozoans that affect humans and animals [4]. As one of the most frequently isolated secondary metabolites from endophytic fungi cultures, cytochalasins are produced by *Phoma* [5], *Hormiscium* [6], *Helminthosporium* [7], *Phomopsis* [8] and *Curualuriu* [9] genera. They have been identified as contaminants of potato [5], tomato [6], pecan [10], rice [11], millet [8] and litchi fruit [9]. The cytochalasins A, B, C, D, and E are highly toxic to the chick, rat, mouse, and guinea pig [11–14] and are teratogenic to both chick and mouse [13, 15–17]. In recent years, most works on endophytic fungi have been centered on plants in the temperate and tropical regions of the world [18].

Plants of the genus *Garcinia* (family Clusiaceae), widely distributed in tropical Africa, Asia, New Caledonia and Polynesia, have yielded an abundance of biologically active and structurally intriguing natural products [19]. *Garcinia* species are known to contain a wide variety of oxygenated and prenylated xanthones, as well as polyisoprenylated benzophenones such as the guttiferones [20].

*Garcinia kola* (Clusiaceae) is a plant of West and Central African origin [21]. In Nigeria, the seed (*Bitter kola*) is chewed for the relief of cough, colds, colic, hoarseness of voice, and throat infections. The plant is also used for the treatment of liver disorders, jaundice, fever, and as a purgative and chewing sticks [21]. We focused on *Garcinia kola* nut because it is one of the most commercialized fruits in West and Central Africa, its highly valued perceived medicinal attributes, and the consumption of large quantities does not cause indigestion. However, several management strategies have been employed for their conservation, but the growth of the molds due to their moisture during that conservation remains a serious problem [22]. Moreover, further studies by Austin [23] attributed the loss of viability of kola nut seeds to reduction in moisture content.

During our investigation, the fungus *Phomopsis* sp*.* associated with that nut was found to be a producer of diverse secondary metabolites, including cytochalasins from its mycelium in potato dextrose agar (PDA) medium. Attracted by the potential production of this class of compounds, a so-called OSMAC (one strain-many compounds) [24] approach was carried out to find compounds. Following the application of the OSMAC principle, we found out that when the culture conditions were changed from PDA medium to solid state medium (rice), fermentation significantly changed and based on high-performance liquid chromatography (HPLC) monitoring, 18-metoxycytochalasin J (1), cytochalasins H (2) and J (3) and alternariol (4) were isolated. In this report, we evaluate the cytotoxic activities of cytochalasins against bacterial species and human cervical cancer cell lines, with emphasis on MDR *Shigella flexneri* and *Vibrio cholerae*.

## Methods

### General experimental procedures

High resolution mass spectra were obtained with an LTQ-Orbitrap Spectrometer (Thermo Fisher, USA) equipped with a HESI-II source. The spectrometer was operated in positive mode (1 spectrum/s; mass range: 100–1000) with nominal mass resolving power of 60 000 at m/z 400 with a scan rate of 1 Hz). It was equipped with automatic gain control to provide high-accuracy mass measurements within 2 ppm deviation using an internal standard; Bis (2-ethylhexyl) phthalate: *m/z* = 391.28428. The spectrometer was attached with an Agilent (Santa Clara, USA) 1200 HPLC system consisting of LC-pump, PDA detector (λ = 260 nm), auto sampler (injection volume 5 μL) and column oven (30 °C). Following parameters were used for experiments: spray voltage 5 kV, capillary temperature 260 °C, tube lens 70 V. Nitrogen was used as a sheath gas (50 arbitrary units) and auxiliary gas (5 arbitrary units). Helium served as the collision gas. The separations were performed by using a Nucleodur C18 Gravity column (50 × 2 mm, 1.8 μm particle size) with a H_2_O (+0.1% HCOOH) (A) / acetonitrile (+0.1% HCOOH) (B) gradient (flow rate 300 μL/min). Samples were analyzed using a gradient program as follows: 80% A isocratic for 1 min, linear gradient to 100% B over 18 min, after 100% B isocratic for 5 min, the system returned to its initial condition (80% A) within 0.5 min, and was equilibrated for 4.5 min. The separation was carried out by preparative HPLC run for 20 min on a Gilson apparatus with UV detection at 220 nm using a Nucleodur C18 Isis column (Macherey-Nagel, Düren, Germany), 5 μm (250 × 16 mm) with a H_2_O (A) / CH_3_OH (B) gradient (flow rate 4 mL/min). Samples were separated by using a gradient program as follows: 60% A and 40% B isocratic for 2 min, linear gradient to 100% B over 18 min, after 100% B isocratic for 5 min, the system returned to its initial condition (60% A) within 0.5 min, and was equilibrated for 4.5 min. The NMR spectra were recorded on a Bruker DRX-500 MHz spectrometer. Chemical shifts (δ) were quoted in parts per million (ppm) from internal standard tetramethylsilane and coupling constants (J) are in Hz. Silica gel [Merck, Kieselgel 60 (0.063–0.200 mm)] was used for column chromatography. Melting points were determined on a BÜCHI melting point b-545 apparatus. UV spectra were measured with the earlier described spectrometer.

### Isolation of endophytic fungus

The fungus was isolated from the nut of *Garcinia kola* bought at Mokolo local market in Yaounde (Cameroon). The plant material was identified at the Cameroon National Herbarium, Yaoundé, where a voucher specimen (N° 27839/SRF-CAM) has been deposited. The seed was first cleaned by washing several times under running tap water and then cut into small slices, followed by successive surface sterilization in 70% ethanol and NaOCl (6-14% active chlorine) for 2 min and finally with sterile distilled water for 2–3 times. The plant material was then dried in between the folds of sterile filter papers and deposited on a Petri dish containing potato dextrose agar medium (PDA) (200 g potato, 20 g dextrose, and 15 g agar in 1 L of H_2_O, supplemented with 100 mg/L of chloramphenicol to suppress bacterial growth). All the plates were incubated at 28 °C to promote the growth of endophytes and were regularly monitored for any microbial growth. On observing the microbial growth, subculturing was done. Each endophytic culture was checked for purity and transferred to freshly prepared PDA plate

### Identification of the fungus CAM240

Cultures were grown on PDA at 25 °C under 12 h light / 12 h darkness cycles. The strain CAM240 formed abundant mycelium that filled out the Petri dishes (9 cm diameter) in 8 days. The isolate was identified by Dr Clovis Douanla-Meli after macroscopic and microscopic examinations of its morphological features. Isolate was deposited as AGMy0319 in the Culture Collection of Federal Research Centre for Cultivated Plants (JKI), Braunschweig, Germany.

### Fungal culture and extraction

*Phomopsis* sp*.* was cultured in 12 flat culture bottles containing 100 g rice and 100 mL water enriched with 0.3% peptone each, autoclaved at 121 °C for 45 min. Each flask received about 5 small pieces of mycelium from PDA plate under sterile conditions. After 40 days of growth at 25 °C, ethyl acetate (12 x 500 mL) was added to each bottle, homogenized and filtered after 24 h and taken to dryness to afford 11.6 g of crude extract.

### Antibacterial assay

#### Microbial growth conditions

A total of six bacterial strains were tested for their susceptibility to compounds and these strains were taken from our laboratory collection (kindly provided by Dr. T. Ramamurthy, NICED, Kolkata). Among the clinical strains of *Vibrio cholerae* used in this study, strains NB2 and SG24 and CO6 belonged to O1 and O139 serotypes, respectively. All these strains were able to produce cholera toxin and hemolysin and multi-drug-resistants (MDR). The other strains used in this study were *V. cholerae* non-O1, non-O139 (strain PC2); and *Shigella flexneri* SDINT. The MDR *V. cholerae* non-O1 and non-O139 strain PC2 isolated from aquatic environment was positive for hemolysin production but negative for cholera toxin production [25]. The American Type Culture Collection (ATCC) strain, *Staphylococcus aureus* ATCC 25923, was used for quality control. The bacterial strains were maintained on agar slant at 4 °C and subcultured on a fresh appropriate agar plates 24 h prior to any antibacterial test. The Mueller Hinton Agar (MHA) was used for the activation of bacteria. The Mueller Hinton Broth (MHB) and nutrient agar (Hi-Media) were used for the MIC and MBC determinations respectively.

### Inocula preparation

Suspensions of bacteria were prepared in MHB from cells arrested during their logarithmic phase growth (4 h) on MHB at 37 °C. The turbidity of the microbial suspension was read spectrophotometrically at 600 nm and adjusted to an OD of 0.1 with MHB, which is equivalent to 1 × 10^8^ CFU/mL. From this prepared solution, other dilutions were made with MHB to yield 1x10^6^ CFU/mL.

### Determination of minimum inhibitory concentration (MIC) and minimum bactericidal concentration (MBC)

MIC and MBC of compounds 1–3 were assessed using the broth microdilution method recommended by the National Committee for Clinical Laboratory Standards [26, 27] with slight modifications. Each test sample was dissolved in dimethylsulfoxide (DMSO) to give a stock solution. The 96-well round bottom sterile plates were prepared by dispensing 180 μL of the inoculated broth (1x10^6^ CFU/mL) into each well. A 20 μL aliquot of the stock solution of compound was added. The concentrations of sample tested were 0.125, 0.25, 0.50, 1, 2, 4, 8, 16, 32, 64, 128, 256 and 512 μg/mL. The final concentration of DMSO in each well was <  1% [preliminary analyses with 1% (v/v) DMSO did not inhibit the growth of the test organisms]. Dilutions of tetracycline and ampicillin served as positive controls, while broth with 20 μL of DMSO was used as negative control. The ATCC strain *Staphylococcus aureus* ATCC 25923 was included for quality assurance purposes. Plates were covered and incubated for 24 h at 37 °C. After incubation, minimum inhibitory concentrations (MIC) were read visually; all wells were plated to nutrient agar (Hi-Media) and incubated. The minimal bactericidal concentration (MBC) was defined as a 99.9% reduction in CFU from the starting inoculums after 24 h incubation interval.

### Cytotoxicity assay

HeLa (Human cervical cancer cell line, ATCC No. CCL-2) and Vero cells (African green monkey kidney cells, normal non-cancer cells, ATCC No. CCL-81), obtained from the American Type Culture Collection (ATCC) were used in this study. Cytotoxic activity was determined using the 3-(4,5-dimethylthiazol-2-yl)-2,5-diphenyltetrazolium bromide (MTT, Sigma, USA) assay reported by Mosmann [28] for the HeLa and Vero cells. This cell viability assay is based on living cell‘s property to transform the MTT dye tetrazolium ring into a purple-colored formazan structure due to the action of mitochondrial and other dehydrogenases inside the cell. The color intensity yielded by the cell population is directly proportional to the number of viable cells, and one can quantify the absorbance measurements using mathematical parameters. Each test sample was dissolved in dimethylsulfoxide (DMSO) to give a stock solution. Compounds 1–3 were prepared from the stock solutions by serial dilution in RPMI 1640 to give a volume of 100 μL in each well of a microtiter plate (96-well). Each well was filled with 100 μL of cells at 2 × 10^5^ cells/mL. The assay for each concentration of compound was performed in triplicates and the culture plates were kept at 37 °C with 5% (v/v) CO_2_ for 24 h. After removing the supernatant of each well and washing twice by PBS, 20 μL of MTT solution (5 mg/mL in PBS) and 100 μL of medium were then introduced. After 4 h of incubation, 100 μL of DMSO were added to each well to dissolve the formazan crystals and the absorbance values at 490 nm were measured with a microplate reader (Bio-RAD 680, USA). The relative cell viability (%) was expressed as a relative percentage of treated cells to the untreated control cells (TC/UC × 100). The rate of cell inhibition was calculated using the following formula: inhibition rate = [1- (OD_test_/OD_negative control_)] × 100%. The LC_50_ values were calculated as the concentration of test sample resulting in a 50% reduction of absorbance compared to untreated cells. Cells treated with 5-fluorouridine + RPMI 1640 served as positive control while cells left untreated + 1% (v/v) DMSO + RPMI 1640 were used as negative control.

### Hemolytic assay

Whole blood (10 mL) from a healthy man was collected into a conical tube containing heparin as an anticoagulant. Erythrocytes were harvested by centrifugation at room temperature for 10 min at 1,000 × *g* and were washed three times in PBS solution. The top layer (plasma) and the next, milky layer (buffy coat with a layer of platelets on top of it) were then carefully aspirated and discarded. The cell pellet was resuspended in 10 mL of PBS solution and mixed by gentle aspiration with a Pasteur pipette. This cell suspension was used immediately.

For the normal human red blood cells, which were in suspension, the cytotoxicity was evaluated as previously described [29]. Compounds 1–3, at concentrations ranging from 32 to 512 μg/mL, were incubated with an equal volume of 1% human red blood cells in phosphate buffered saline (10 mM PBS, pH 7.4) at 37 °C for 1 h. Tetracycline was tested simultaneously. Non-hemolytic and 100% hemolytic controls were the buffer alone and the buffer containing 1% Triton X-100, respectively. Cell lysis was monitored by measuring the release of hemoglobin at 595 nm with a spectrophotometer (Thermo Scientific, USA). Percent hemolysis was calculated as follows: [(*A595* of sample treated with compound - *A*595 of sample treated with buffer)/(*A*595 of sample treated with Triton X-100 – *A595* of sample treated with buffer)] x 100.

### Statistical analysis

Statistical analysis was carried out using Statistical Package for Social Science (SPSS, version 12.0). The experimental results were expressed as the mean ± Standard Deviation (SD). Group comparisons were performed using One Way ANOVA followed by Waller-Duncan Post Hoc test. A p value of 0.05 was considered statistically significant.

## Results and discussion

### Identification of the fungus

**Fig. 1:**
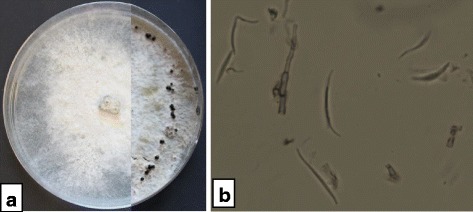
Macroscopic (**a**) and microscopic (**b**) aspects of *Phomopsis* sp

Macroscopic examination of the isolate revealed that colonies were cottony, developing compact aerial mycelium, at first uniformly white (Fig. 1a) then becoming whitish with pale brown patches. The reverse side of cultures was whitish, then turned light brown with scattered darker spots which later appeared regularly concentrical. Conidiation began in 12-day old colonies with the formation of spherical, subglobose to ampuliform black stromata, measuring 210–250 × 220–380 μm and arranged in a circle in the Petri disch (Fig. 1a) and containing pycnidia. Watery exudate drops from pycnidia contained only beta conidia. These were 17–28.5 × 0.9-1.9 μm, unicellular, hyaline, filiform and mostly slightly curved at one end (Fig. 1b).

Cultural and morphological features of the strain CAM240 enabled its reliable placement in the genus *Phomopsis*. There was noticeable morphological similarity with *Phomopsis longicolla* [30], a species generally known as a Soybean pathogen, but that can be isolated as endophyte from other different host plants. With reference to recent revision of species concept in *Phomopsis*, specific determination requires a muli-locus analysis of ITS, tef and ß-tubulin loci [31]. Therefore, taxonomy of strain CAM240 as based only on the morphology in this study was restricted to generic level.

### Chemical analysis

**Fig. 2:**
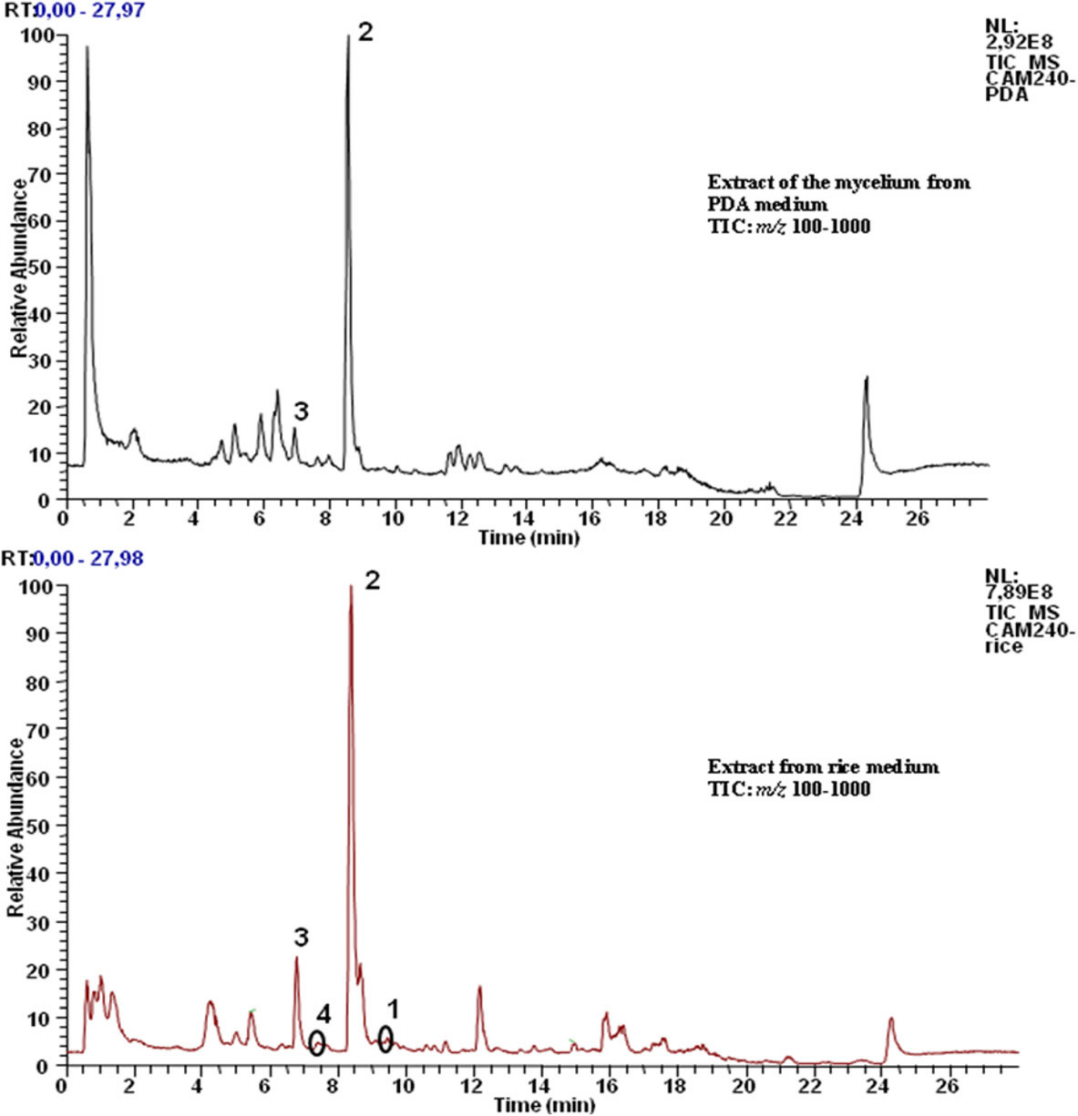
HR-LC-MS chromatograms of the mycelium from PDA and rice media extracts

**Fig. 3:**
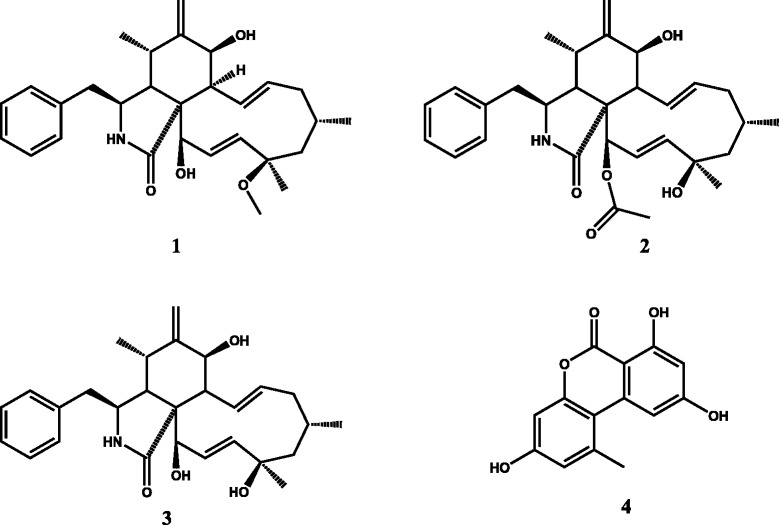
Chemical structures of compounds 1–4 from *Phomopsis* sp*.* 1: 18-metoxycytochalasin J; 2: cytochalasin H; 3: cytochalasin J and 4: alternariol

The mycelium from Petri dish after ten days fermentation was extracted with 10 mL ethyl acetate. The obtained extract was submitted to HR-LC-MS and the major compounds were directly identified (Fig. 2). The crude extract (11.60 g) from the large scale fermentation was firstly submitted to HR-LC-MS and then chromatographed on a silica gel column (0.04–0.063 mm, 6 cm x 60 cm, 100 g) eluting with cyclohexane, mixture cyclohexane/ethyl acetate by increasing the polarity and finally with methanol. 56 fractions of 200 mL each were collected and combined according to TLC profile into 17 fractions. Each fraction was monitored by LC-MS and fractions 7, 10 and 16 were further purified by means of high performance reverse phase liquid chromatography to yield 3 cytochalasins: 18-metoxycytochalasin J (1) (4.1 mg, t_R =_ 9.48 min) isolated as brown amorphous powder, its molecular formula was determined to be C_29_H_39_O_4_N by its HRESIMS *m/z* 466.29587 [M + H]^+^ (calculated 466.29573 for [M + H]^+^) [32]; cytochalasin H (2) (136.2 mg, t_R =_ 8.94 min), isolated as white powder, HRESIMS *m/z* 494.28949 [M + H]^+^ (calculated for C_30_H_40_O_5_N, 494.29065) [33]; cytochalasin J (3) (16.7 mg, t_R =_ 7.41 min) was obtained as white crystals, HRESIMS *m/z* 452.28052 [M + H]^+^ (calculated for C_28_H_38_O_4_N, 452.28008) [34], was the major metabolite and alternariol (4) (5.3 mg, t_R =_ 7.55 min) as a white powder, HRESIMS *m/z* 259.06009 [M + H]^+^ (calculated for C_14_H_11_O_5,_ 259.06065) [35]. The chemical structures of the isolated compounds are shown in Fig. 3.

The chemical investigation of the crude extract from the rice medium of *Phomopsis* sp*.* harboring nut of *Garcinia kola*, by means of different chromatography techniques yielded four main compounds. Cytochalasins were the major secondary metabolites as detected and shown in Fig. 2, and this class of compounds is commonly found in *Phomopsis* genus.

### Antibacterial activity

The cytochalasins showed different degrees of antibacterial activities against the tested bacterial pathogens (Table 1). *Shigella flexneri* SDINT was the most sensitive microorganism while *Vibrio cholerae* SG24 and *V. cholerae* PC2 were the most resistant. Ampicillin did not show any antibacterial activity against *V. cholerae* NB2, *V. cholerae* PC2, and *Shigella flexneri* SDINT at concentrations up to 512 μg/mL while these multi-drug resistant bacterial strains were found sensitive to the cytochalasin metabolites. This finding suggests the antibacterial potencies of these compounds in particular for the treatment of multi-drug-resistant (MDR) bacterial strains. Compounds 1, 2 and 3 showed selective activities; their inhibitory effects being noted respectively on 4/6 (66.66%), 5/6 (83.33%) and 4/6 (66.66%) of the studied microorganisms. A keen look at the MBC values indicates that most of them are equal to their corresponding MICs. This proves that the killing effects of many tested samples could be expected on the sensitive strains [36].

**Table 1:** Inhibition parameters (MIC, MBC) of compounds 1–3 and reference antibacterial drugs

Antibacterial activity (MIC and MBC in μg/mL)							
Compounds	Inhibition parameters	*V. cholerae* SG24	*V. cholerae* CO6	*V. cholerae* NB2	*V. cholerae* PC2	*S. flexneri* SDINT	*S. aureus* ATCC 25923
1	MIC	>512	512	512	>512	128	128
	MBC	>512	512	512	>512	128	128
	MBC/MIC	/	1	1	/	1	1
2	MIC	>512	512	512	256	128	256
	MBC	>512	512	512	256	128	256
	MBC/MIC	/	1	1	1	1	1
3	MIC	>512	512	512	>512	128	512
	MBC	>512	512	512	>512	128	512
	MBC/MIC	/	1	1	/	1	1
Tetracycline	MIC	0.50	2	0.50	0.50	16	16
	MBC	1	8	2	1	32	32
	MBC/MIC	2	4	4	2	2	2
Ampicillin	MIC	16	16	>512	>512	>512	16
	MBC	16	16	>512	>512	>512	16
	MBC/MIC	1	1	/	/	/	1

/ not determined; *MIC* Minimum Inhibitory Concentration; *MBC* Minimum Bactericidal Concentration

The present study showed significant antibacterial activity of cytochalasin compounds against MDR entero-pathogenic bacteria including the clinical isolates of toxigenic *Vibrio cholerae*, the causative agents of dreadful disease cholera and *Shigella* sp., the causative agent of shigellosis. These compounds were having significant antibacterial activities against Gram-positive bacterium, *Staphylococcus aureus*. Although cytochalasin compounds have been reported to possess interesting activity against a wide range of microorganisms [37], no study has been reported on the activity of the metoxycytochalasin J (1), cytochalasins H (2) and J (3) against these types of pathogenic strains.

### Cytotoxicity activity

Compounds 1–3 were evaluated for their anticancer against human cervical cancer cells (HeLa cells) (Table 2). The lowest LC_50_ value (corresponding to the most cytotoxic compound) was found with compound 3 (LC_50_ = 3.66 μg/mL) followed in decreasing order by compound 1 (LC_50_ = 8.18 μg/mL) and compound 2 (LC_50_ = 35.69 μg/mL) (Table 2). Interestingly, the cytotoxicity of compound 3 can be considered more important when taking into consideration the criterion of the American National Cancer Institute (NCI) regarding the cytotoxicity of pure compounds (LC_50_ < 4 μg/mL) [38]. The data also showed that the tested compounds were most cytotoxic to HeLa cells (LC_50_ = 3.66–35.69 μg/mL) when compared with Vero cells (LC_50_ = 73.88–129.10 μg/mL) indicating that they are less toxic to normal cells. Our results are in agreement with those of Xu et al. [39] who showed the cytotoxicity activity of some cytochalasin compounds isolated from the solid substrate culture of *Endothia gyrosa IFB-E023* against the human leukaemia K562 cell line with the IC_50_ values varying between 1.5 to 28.3 μM.

**Table 2:** Cytotoxicity (LC_50_ in μg/mL) of compounds 1–3 and their selectivity index (SI)

Compounds	Cytotoxicty (LC_50_)		Selectivity Index*						
	HeLa cells	Vero cells	HeLa cells	*V. cholerae* SG24	*V. cholerae* CO6	*V. cholerae* NB2	*V. cholerae* PC2	*S. flexneri* SDINT	*S. aureus* ATCC 25923
1	8.18 ± 0.92^a^	93.02 ± 2.54^a^	11.37	/	0.18	0.18	/	0.72	0.72
2	35.69 ± 1.31^b^	129.10 ± 1.20^b^	3.61	/	0.25	0.25	0.50	1.00	0.50
3	3.66 ± 0.33^c^	73.88 ± 0.92^c^	20.18	/	0.14	0.14	/	0.57	0.14
5-FU	0.25 ± 0.05^d^	4.63 ± 0.17^d^	18.52	/	/	/	/	/	/

/: not determined; *5-FU* 5-Fluorouridine; *SI* LC_50_ on Vero cells /MIC or LC_50_ on HeLa cells; *: SI obtained from average MIC. In the same column, LC_50_ value marked with different superscript letters (a, b, c, d) are significantly different (*p* < 0.05)

In the present study, Selectivity Index (SI) of active compounds was determined in order to investigate whether the cytotoxic activity was specific to cancer cells/bacterial strains. The SI of the samples are defined as the ratio of cytotoxicity (LC_50_ values) on normal non-cancer cells (Vero cells) to cancer cells (HeLa cells) or bacterial cells: SI = LC_50_ on Vero cells / LC_50_ on HeLa cells or MIC. Test agents with SI equal or higher than ten were considered to have high selectivity towards cancer cells [40]. Apart from compounds 1 and 3 on HeLa cells, the SI values of the tested samples against the HeLa cells and bacterial strains ranged from 0.14 to 3.61 and could be considered as poor.

### Hemolytic activity

**Fig. 4:**
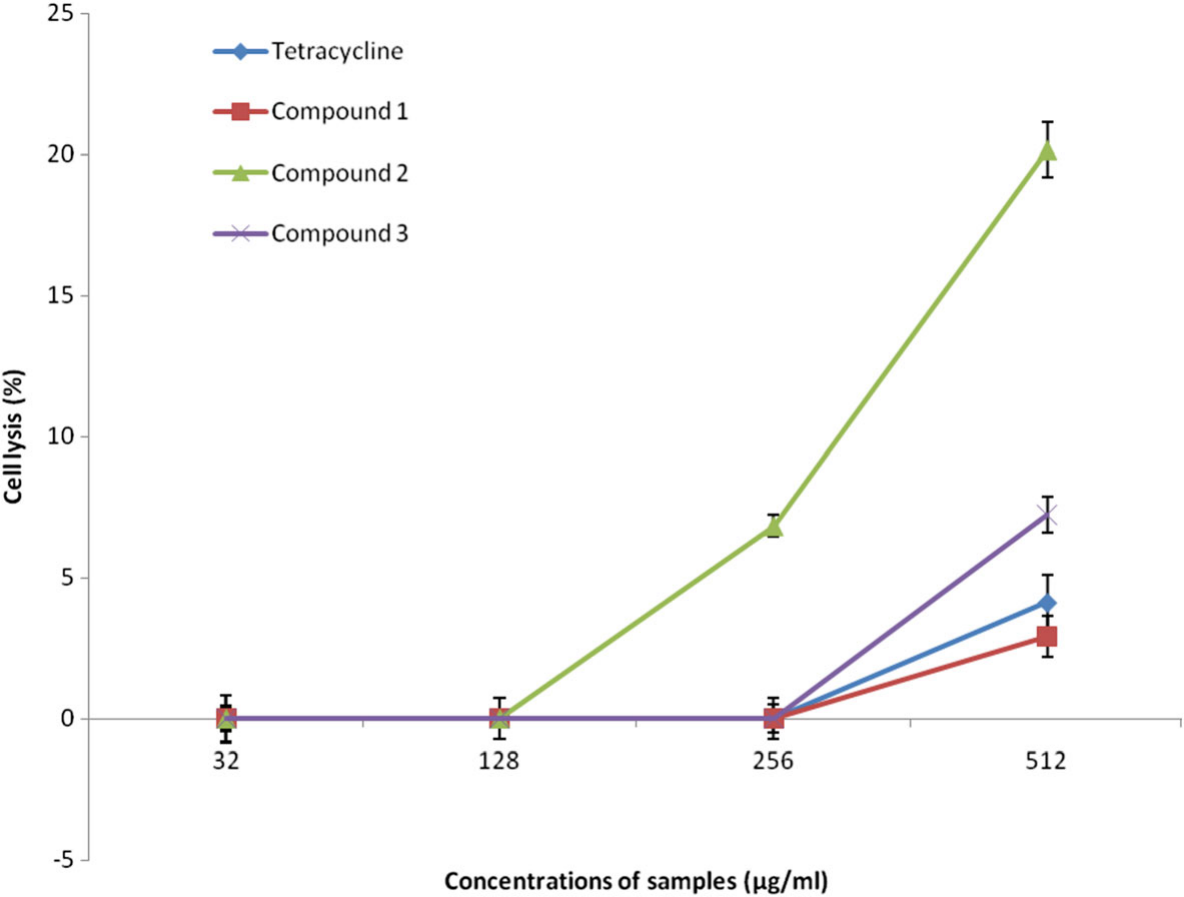
Cytotoxicity of compounds 1–3 against red blood cells. The data are means of triplicate experiments

Human red blood cells provide a handy tool for toxicity studies of compounds, because they are readily available, their membrane properties are well known, and their lysis is easy to monitor by measuring the release of hemoglobin [29]. The hemolytic activities of compounds 1–3, and tetracycline on human red blood cells (as a function of sample concentration) are shown in Fig. 4. At the highest concentration tested in this study (512 μg/mL), compounds 1, 3 and tetracycline caused less than 10% hemolysis, while compound 2 caused 20.14% hemolysis.

## Conclusions

The chemical study of the ethyl acetate extract of *Phomopsis* sp*.* mycelium afforded three known cytochalasins including 18-metoxycytochalasin J (1), cytochalasins H (2) and J (3) together with alternariol (4). Compounds 1, 2 and 3 showed different degrees of antibacterial activities against MDR clinical strains of enteropathogenic bacteria with low toxicity to human red blood cells and normal Vero cells. These compounds also showed significant cytotoxic properties against human cervical cancer cells. The overall results of this study indicate that cytochalasin compounds 1–3 isolated from the *Phomopsis* sp*.* mycelium could be a clinically useful alternative for the treatment of cervical cancer and severe infections in particular those caused by *Shigella flexneri* and *Vibrio cholerae* strains resistant to ampicillin.

## Abbreviations

5-FU: 5-Fluorouridine
ATCC: American Type Culture Collection
DMSO: Dimethylsulfoxide
HMBC: Heteronuclear Multiple Bond Connectivities
HPLC: High Performance Liquid Chromatography
HR-ESIMS: High-resolution electrospray ionization mass spectrometry
HR-LC-MS: High-resolution-liquid chromatography–mass spectrometry
IR: Infra-red
LC_50_: Concentration of test sample resulting in a 50% reduction of absorbance compared to untreated cells
LC-MS: Liquid chromatography–mass spectrometry
MBC: Minimum bactericidal concentration
MDR: Multi-drug-resistant
MHA: Mueller Hinton agar
MHB: Mueller Hinton broth
MIC: Minimum inhibitory concentration
MTT: 3-(4,5-dimethylthiazol-2-yl)-2,5-diphenyltetrazolium bromide
NA: Nutrient agar
NMR: Nuclear Magnetic Resonance
PBS: Phosphate buffered saline
PDA: Potato dextrose agar
RPMI: Roswell Park Memorial Institute
SI: Selectivity Index
TLC: Thin layer chromatography
UV: Ultra violet
